# MEMS Based Broadband Piezoelectric Ultrasonic Energy Harvester (PUEH) for Enabling Self-Powered Implantable Biomedical Devices

**DOI:** 10.1038/srep24946

**Published:** 2016-04-26

**Authors:** Qiongfeng Shi, Tao Wang, Chengkuo Lee

**Affiliations:** 1 Department of Electrical and Computer Engineering, National University of Singapore, 4 Engineering Drive 3, 117576 Singapore; 2 Center for Intelligent Sensors and MEMS, National University of Singapore, 4 Engineering Drive 3, 117576 Singapore

## Abstract

Acoustic energy transfer is a promising energy harvesting technology candidate for implantable biomedical devices. However, it does not show competitive strength for enabling self-powered implantable biomedical devices due to two issues – large size of bulk piezoelectric ultrasound transducers and output power fluctuation with transferred distance due to standing wave. Here we report a microelectromechanical systems (MEMS) based broadband piezoelectric ultrasonic energy harvester (PUEH) to enable self-powered implantable biomedical devices. The PUEH is a microfabricated lead zirconate titanate (PZT) diaphragm array and has wide operation bandwidth. By adjusting frequency of the input ultrasound wave within the operation bandwidth, standing wave effect can be minimized for any given distances. For example, at 1 cm distance, power density can be increased from 0.59 μW/cm^2^ to 3.75 μW/cm^2^ at input ultrasound intensity of 1 mW/cm^2^ when frequency changes from 250 to 240 kHz. Due to the difference of human body and manual surgical process, distance fluctuation for implantable biomedical devices is unavoidable and it strongly affects the coupling efficiency. This issue can be overcome by performing frequency adjustment of the PUEH. The proposed PUEH shows great potential to be integrated on an implanted biomedical device chip as power source for various applications.

Implantable biomedical devices are widely used to improve the life quality of millions of patients in various scenarios, such as pacemakers, cardiac defibrillators, neural stimulators, blood pressure monitors, etc^1^,^2^. Nowadays conventional implantable biomedical devices require power supply from batteries inside body. New emerging devices may require small power supply, such as devices for activities like cardiac sensing, adaptive pacing and programmability only demand operation power of 0.3 μW^3^,^4^. But a commercialized pacemaker still requires sizable operation power from a few microwatts to 20 μW. Even though size and capacity of batteries have been greatly improved in the past few years, lifetime of them still remains limited. Furthermore, volume of a battery typically takes up 2/3 volume of the whole pacemaker. Depleted batteries need to be replaced by surgical procedures which increase hospitalization time and expose the patients to health risks such as high morbidity or even mortality. Different energy harvesting technologies are developed in order to extend the lifetime of batteries or even to replace the batteries. There are two major approaches reported to harvest energy aiming at potential applications of implantable biomedical devices. The first method is harvesting energy from periodical vibration of heart or lung by implantable triboelectric devices^5^,^6^,^7^,^8^,^9^ or piezoelectric devices such as ZnO devices^10^,^11^,^12^,^13^, lead zirconate titanate (PZT) devices^14^,^15^,^16^,^17^,^18^. Triboelectric energy harvesters are based on the contact electrification phenomenon of two different materials, which results in triboelectric charges and current flowing in external circuit as they are put in contact with each other and separated^19^,^20^,^21^. Piezoelectric energy harvesters are based on the generation of internal electrical charges and potential in a piezoelectric material when subjected mechanical force, known as the piezoelectric effect. A variety of piezoelectric energy harvesters are developed, working in either d_31_ mode (direction of applied stress and generated voltage is perpendicular)^22^,^23^ or d_33_ mode (direction of applied stress and generated voltage is the same)^24^. Z. L. Wang *et al*. reports a ZnO nanowire array based nanogenerator for the first time to harvest mechanical energy^10^, but the output of the device is quite limited for practical application due to the low piezoelectric constant of the ZnO nanowires. A flexible PZT ribbon energy harvester on polyimide substrate is reported by C. Dagdeviren *et al*. to harvest energy from motions of animal hearts, lungs and diaphragms^14^, because PZT has 20 times higher piezoelectric coefficient than ZnO. Recently Q. Zheng *et al*. has reported a breathing-driven implanted triboelectric energy harvester to *in vivo* power a pacemaker^5^. But performance of triboelectric energy harvesters is greatly affected by the implanted environment and decreases significantly when *in vivo* data are compared to *in vitro* results. These implantable energy harvesters using vibration associated with activities of internal organs face a common limitation that output power is highly dependent on organ size, shape and usage condition^14^. Energy harvesting from small animals like rabbit or rat will not generate sound output power. Therefore, the adaptability and stability of reported implantable energy harvesters need to be improved a lot in order to make these novel power sources become practical in various implantable biomedical usage scenarios.

The second energy harvesting approach is to convey energy from external source outside body through propagating wave like inductive power transfer (IPT)^25^,^26^,^27^ and acoustic energy transfer (AET)^28^,^29^,^30^,^31^. Obviously harvesting energy from outside source provides adaptable and stable output power irrelevant of animal size, implanted location, organ shape, or organ vibration. Conventional energy transfer techniques to transmit energy to an implantable energy harvester include IPT^32^,^33^ and AET^34^,^35^. In the IPT or AET system, an energy source (transmitter) is used to transfer energy and an energy receiver (energy harvester) is used to collect the arrived energy. IPT is highly investigated and developed by researchers due to simple device configuration and good performance, *e.g.* recent reported IPT systems are able to transfer energy with high efficiency up to 90%^36^,^37^. However, drawbacks of IPT are also significant, for example, significantly decreased efficiency as distance increases, adverse side effects on human tissue, interference with integrated electrical circuits, the electromagnetic wave used in IPT can not pass through conductive media such as the metal housing of a pacemaker. These issues have greatly limited the practical applications of IPT^38^,^39^. AET is an emerging technology which receives increasing attention due to its advantages over IPT^34^,^35^,^39^. AET can transfer energy over a relative long distance even when a conductive medium is present^40^. There is no adverse side effect to human tissue which makes it ideally suitable for implantable applications. M. G. L. Rose *et al*. reports an AET system that is able to transfer energy over a distance as long as 10 cm^41^. Another AET system with high output performance is reported by S. Ozeri *et al*. based on a 15 mm diameter disc shape PZT element^42^. An ultrasonically powered implantable micro-oxygen generator (IMOG) proposed by T. Maleki *et al*. is demonstrated for *in situ* tumor oxygenation^28^. P. J. Larson *et al*. reports a cylindrical bulk PZT element integrated with diode, capacitor and neural electrode for implantable nerve cuff stimulation^29^. Implantable energy harvesters aim at providing energy to self-powered implantable systems. Hence miniaturized size of implantable energy harvester facilitating integration with a biomedical device is always preferable. But conventional AET energy harvesters use bulk PZT transducers with size from millimeters to centimeters^30^,^31^,^41^,^42^,^43^,^44^,^45^. The large size of bulk PZT transducers greatly limits the integration capability and increases the total size of the final integration system. Even though piezoelectric micromachined ultrasonic transducer (pMUT) has been reported as sensors and actuators^46^,^47^,^48^, there is no reported AET data of pMUT. Furthermore, ultrasonic wave propagating between two transducers is reflected back and forth by the transducer surface, leading to standing wave between the ultrasound transmitter and receiver. For each transfer frequency, power and efficiency fluctuate significantly with peaks and valleys when increasing the distance between the transmitter and receiver. In practical usage of implanted biomedical devices, implanted depth from skin surface varies upon body size of patients and could not be accurately controlled in the surgical operation process. Thus the transferred power may falls into a minimum at a given distance due to the standing wave effect, resulting in very low efficiency. Frequency adjustment technology is developed in this study to overcome this issue. When the ultrasound frequency is changed, ultrasound wavelength changes accordingly and thus the position of peaks and valleys in the standing wave also varies. Therefore, by adjusting the transfer frequency, a maximum power point can be achieved for any given distances. To realize the frequency adjustment function, it requires a broadband energy harvester. Otherwise, changing the frequency itself will cause power reduced dramatically if an energy harvester of narrow operation bandwidth is used. Unfortunately, conventional bulk PZT transducers normally have limited bandwidth due to large impedance mismatch between the transducers and medium. Therefore, changing operation frequency of bulk PZT transducers will significantly decrease transferred power and efficiency.

In this paper, we propose a new microelectromechanical systems (MEMS) based broadband piezoelectric ultrasonic energy harvester (PUEH) aiming at extending the battery lifetime of a pacemaker or other implantable biomedical devices. Figure 1(a) shows the concept of the proposed PUEH driving a pacemaker inside of human body. The PUEH is fabricated from miniaturized PZT diaphragm array, making it ideally suitable to be integrated with an implantable pacemaker without significantly increasing the device volume. The PUEH can harvest ultrasonic energy from an external ultrasound head that is commonly used in hospitals for diagnostic application, while it is impossible for IPT passing through the metal housing of pacemaker. Due to the optimized design of the thin PZT diaphragm, two resonant modes are overlapped, providing an extra wide operation bandwidth. Standing wave induced frequency loss can be overcome by adjusting ultrasound frequency for any given implanted distances. For example, at 1 cm distance in water, the output power density can be increased from 0.59 μW/cm^2^ to 3.75 μW/cm^2^ at ultrasound intensity of 1 mW/cm^2^ by changing the frequency from 250 kHz to 240 kHz. Distance fluctuation during energy transfer process caused by breathing or muscle movement also can be overcome by this frequency adjustment. When the distance slightly deviates (*i.e.* from 1 cm to 1.1 cm) at 370 kHz, the output power density largely drops from 4.10 μW/cm^2^ to 0.18 μW/cm^2^. Such power and efficiency loss can be retrieved as 4.06 μW/cm^2^ by adjusting the ultrasound frequency to 340 kHz. The proposed MEMS based broadband PUEH shows great potential as a power source used in various implantable biomedical devices for diversified applications.

## Results

### Design

**Figure 1:**
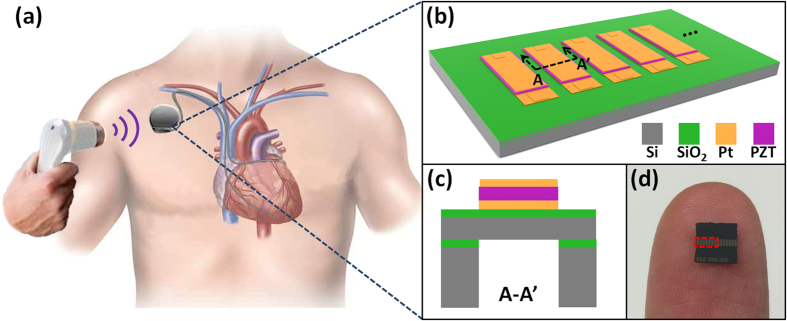
(**a**) Schematic of the proposed PUEH integrated with a pacemaker and powered by an ultrasound head. (**b**) Schematic illustration of the proposed PUEH. (**c**) Cross-sectional view of the proposed PUEH. (**d**) Photograph of the PUEH on a human finger.

**Figure 2:**
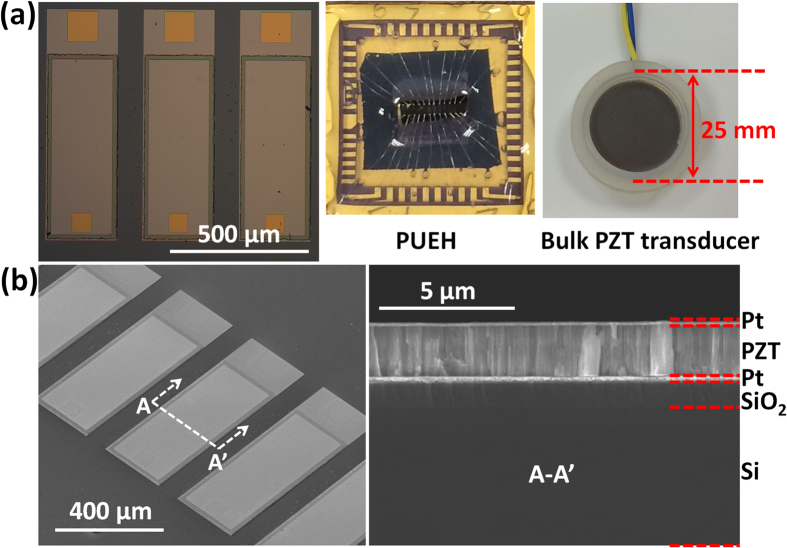
(**a**) Optical microscope image of the PZT diaphragm array (left), photograph of the PUEH on a DIP packaging with wirebonding (middle) and photograph of a bulk PZT transducer used as a transmitter in AET system (right). (**b**) Bird view and cross-sectional view SEM images of the PUEH.

The overall device structure of the PUEH is shown in Fig. 1(b). The PUEH consists of 7 PZT diaphragms that are connected in parallel to increase the output current and power. PZT diaphragm array is fabricated on a silicon on insulator (SOI) wafer. Dimension of each PZT diaphragm is 500 μm × 250 μm with multilayered structure containing Si, SiO_2_, bottom Pt electrode, PZT, and top Pt electrode as shown in Fig. 1(c). The PZT diaphragm with optimized length to width ratio and thickness provides broadband property without compromising device performance and robustness. A dicing chip with size of 5 mm × 5 mm held by a human finger is shown in Fig. 1(d). The spacing between two PZT diaphragms is 90 μm. Effective area of the PUEH is 2.058 mm^2^, indicated by the red dash rectangle in Fig. 1(d). Benefited from the micro fabrication of the miniaturized PZT diaphragms, array structure on one single chip to improve output performance is simple to achieve, as well as the integration with other functioning electrical sensors, stimulators, etc. Fig. 2(a) illustrates, from left to right, the optical microscope image of the PZT diaphragms, photograph of PUEH packaged on dual in-line package (DIP) and a commercialized bulk PZT transducer (M165D25, Pro-wave corp.) which is used as a transmitter in the AET system. Figure 2(b) shows the scanning electron microscope (SEM) images of PZT diaphragm patterns and cross-sectional view of multilayered structure of the diaphragm.

### Characterization

**Figure 3:**
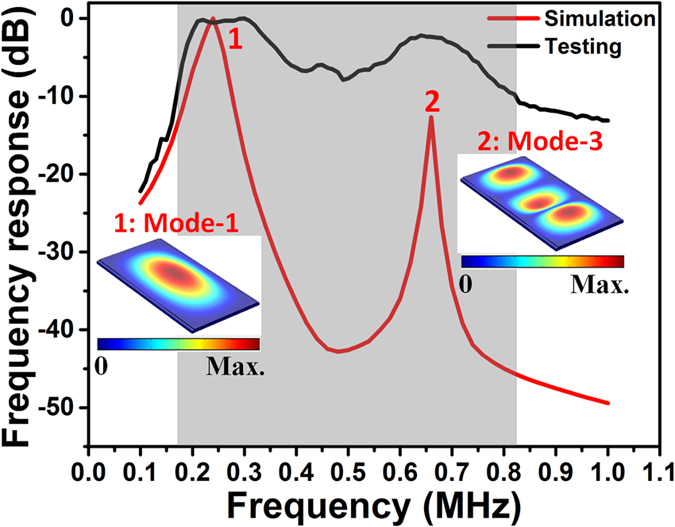
Frequency response of the PUEH from simulation and testing results.

In order to find out the resonant frequency of the PUEH, it is first immersed into a water tank and connected to a signal generator. Then a burst-mode sinusoidal signal is applied to excite the PUEH to generate ultrasound in water medium. Next, a hydrophone (2118, Precision Acoustic Ltd) is utilized to record the generated ultrasound pressure at 3 cm distance. The burst-mode sinusoidal signal is used to eliminate the reflection signal between PUEH and hydrophone. Frequency of the sinusoidal signal is changed from 100 kHz to 1 MHz to obtain the frequency response of the PUEH, shown in Fig. 3. A finite element analysis (FEA) model is built by using COMSOL Multiphysics to simulate the resonant frequency of the PUEH. The testing result matches quite well with the simulation, as illustrated in Fig. 3. The relatively thin diaphragm enables a better acoustic impedance match with the medium, and therefore a larger frequency bandwidth. Moreover, because of the optimized diaphragm length to width ratio, the 1st and 3rd resonant mode are overlapped, resulting in an extra-wide operation bandwidth of the PUEH. The PUEH has -6 dB bandwidth of 73.7% with central frequency of 285 kHz for 1st resonant mode and -6 dB bandwidth of 30.8% with central frequency of 650 kHz for 3rd resonant mode. For -10 dB frequency response, it covers two resonant modes within a frequency range from 170 kHz to 820 kHz (650 kHz), showing the excellent broadband property of the PUEH. Frequency can be adjusted in this range without losing the energy harvesting capability and efficiency.

**Figure 4:**
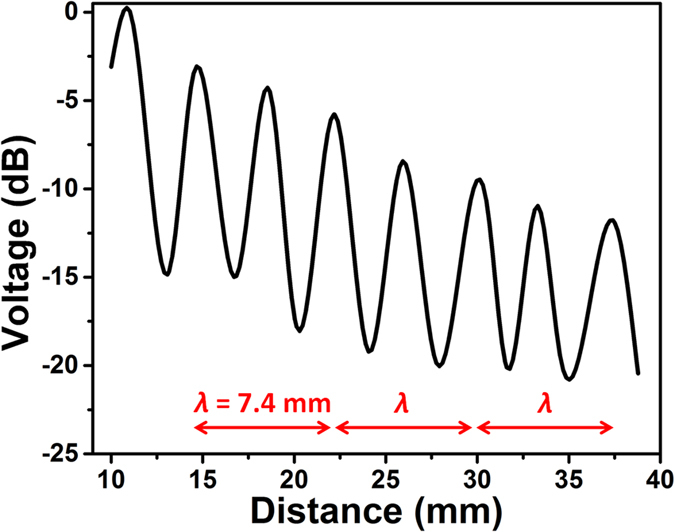
Measured standing wave effect between two PUEHs.

**Figure 5:**
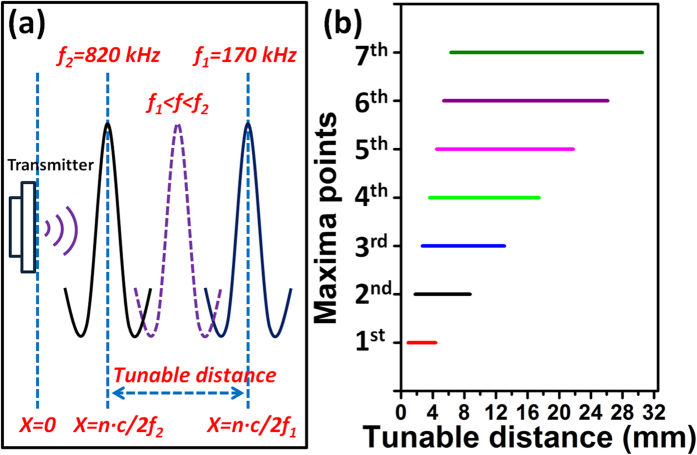
(**a**) Schematic of tunable distance for a maxima point. (**b**) Tunable distance range for first 7 maxima points.

When an AET system is operated in a certain medium, ultrasonic waves reflect back and forth between the surfaces of transmitter and receiver. The standing wave is formed as the reflected waves of different orders interfere with the original ultrasonic wave. Figure 4 shows a typical standing wave effect when one PUEH is used as transmitter and another PUEH is used as receiver in water medium. The reason why the bulk PZT transducer is not used as transmitter here is because the distance variation here is within the near field region of the bulk PZT, but the near field region for PUEH is within 100 μm (supplementary information, Figure S1). Hence in order to eliminate the interference of near field, two PUEHs are used to form an AET system to measure the standing wave. Sinusoidal with 10 Vpp amplitude and 200 kHz frequency is applied to the transmitter. Distance between the transmitter and receiver is increased from 1 cm to 4 cm. It can be observed from Fig. 5 that when the distance increases, maxima voltage points and minima voltage points occur alternatively. The maxima voltage points occur at the distance given by following equations:


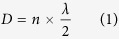



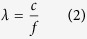


where 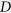 is the distance between the transmitter and receiver, 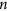 is the positive integer (1, 2, 3, …), 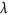 is the wavelength in medium, 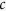 is the speed of sound in medium (1480 m/s in water) and 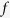 is frequency. When the distance is a multiple of half wavelength of the ultrasonic wave, the reflected ultrasonic wave is in phase with the original ultrasonic wave, thus the voltage at that distance is enhanced. Minima voltage points occur at a distance in the middle of two maxima voltage points. Since the frequency used here is 200 kHz, ultrasound wavelength is 7.4 mm in water based on equation (2). Therefore, when the distance is a multiple of 3.7 mm, there is a maxima voltage point. It is worth noting that the values of maxima voltage points decrease exponentially when increasing distance, due to the diffraction of the ultrasonic wave from transmitter.

Benefited from the broadband property of the PUEH, frequency can be changed within the wide operation bandwidth without losing the transduction efficiency. When frequency changes, locations of maxima voltage points also change accordingly based on equation (1) and (2). Thus when frequency is changed within the operation bandwidth range, locations of maxima voltage points also change within a tunable distance range. Bandwidth of the PUEH is from 170 kHz (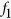) to 820 kHz ( *f*_2_), then location of 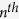 maxima voltage point covers distance range from 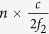 to 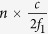 with respect to the transmitter. The tunable distance range for 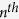 maxima voltage point is indicated in Fig. 5. Figure 5(a) shows the schematic of the maxima voltage point changing with varying the frequency. Figure 5(b) shows the tunable distance range for the first 7 maxima voltage points. The details of tunable distance range are also listed in Table 1. If a given distance between the transmitter and receiver is within the tunable distance range of 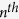 maxima voltage point, the 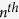 maxima voltage point can always be located to that given distance by adjusting frequency within the operation bandwidth of PUEH. For example, if the distance between the transmitter and receiver is 5 mm, from Table 1 we can see that it falls in the tunable distance range of the 2nd, 3rd, 4th and 5th maxima voltage point. When performing the frequency adjustment at 5 mm, there are 4 maxima voltage points that can be achieved for 4 different frequencies within the operation bandwidth.

### Energy harvesting evaluation

**Figure 6:**
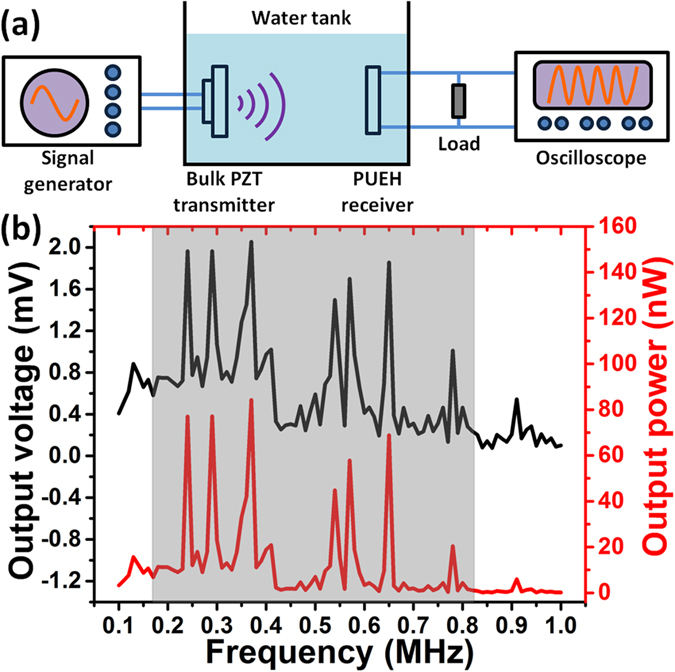
(**a**) Schematic drawing of testing setup for voltage and power measurement. (**b**) Voltage and power when ultrasound intensity of 1 mW/cm^2^ is applied.

In a practical implantation case, the implanted depth of a biomedical device is normally different due to the difference of human body and manual surgical process. There is a high chance that the distance (implanted depth) may fall at the location of a minima point, resulting in poor output and low transduction efficiency. This problem has not been fully addressed, and the researchers usually only demonstrate the highest efficiency at a precise distance. This is actually not available for practical applications. Therefore, we try to overcome this problem by performing frequency adjustment with the broadband PUEH. To demonstrate the energy harvesting capability of the broadband PUEH for a given distance, an alternative AET system is built by a bulk PZT transmitter and a PUEH receiver in a water tank as depicted in Fig. 6(a). Photograph of testing setup is shown in supplementary information, Figure S2. The bulk PZT and PUEH are both covered by grounded Al foil with a window open for ultrasound propagation to eliminate the electromagnetic coupling between devices. Both the bulk PZT and the PUEH are immersed under DI water and the distance is fixed to 1 cm. The impedances of the PUEH and bulk PZT measured in water with respect to frequency are shown in supplementary, Figure S3. The PUEH is connected to a 50Ω resistor load, while the bulk PZT is excited by a signal generator with 10 Vpp sinusoidal signals. The output voltage on the resistor load is measured by an oscilloscope.

For different frequency (from 100 kHz to 1 MHz), the generated ultrasound pressure and thus ultrasound intensity from the bulk PZT transmitter are different. In order to eliminate the effect of bulk PZT transmitter and provide a fair comparison between different frequency for PUEH, output voltage and power are normalized to the magnitude when ultrasound intensity of 1 mW/cm^2^ is applied. Ultrasound pressure can be measured by a hydrophone placed at 1 cm distance from the bulk PZT, and then ultrasound intensity can be calculated by the following equation:


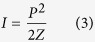


where 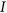 is the ultrasound intensity, 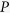 is the ultrasound pressure and 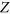 is the acoustic impedance (for water medium, 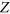 is 1.48 × 10^6^ kg/m^2^·s). Figure S4(a,b) in supplementary information show the ultrasound pressure of the bulk PZT from COMSOL Multiphysics simulation and hydrophone measurement, respectively. It can be observed that the testing data matches with the simulation data. At 1 cm distance, ultrasound pressure from the bulk PZT varies a lot when changing the frequency.

The output voltage and power of the PUEH on 50 Ω resistor load are shown in Fig. 6(b). Both the output voltage and power are normalized to 1 mW/cm^2^ input ultrasound intensity. For the 1 cm transfer distance, power peaks and valleys occur alternately when frequency is increased from 100 kHz to 1 MHz. Locations of these peaks are the effect of standing wave given by equation (1), but the magnitudes are the effect of both standing wave and PUEH frequency response property. Due to standing wave effect, the magnitude of lower order maxima point is larger than higher order one as shown in Fig. 4. But, due to PUEH frequency response property, the magnitude of maxima points should follow the trend of the frequency response curve. The voltage and power curves in Fig. 6(b) are the effect of these two factors. Within the bandwidth of PUEH, standing wave effect can be minimized by adjusting the transfer frequency. For example, if we perform energy transfer at the 1st resonant frequency of the PUEH (250 kHz), normalized output power is only 12.0 nW. Then if frequency adjustment is applied, just by changing the frequency to 240 kHz, normalized output power can be increased to 77.1 nW which is more than 6 times higher. The highest normalized power and power density of the PUEH is 84.3 nW and 4.1 μW/cm^2^, respectively, when frequency is 370 kHz. This is because for 370 kHz, 1 cm distance is the distance that satisfies maxima point and thus power is enhanced. Furthermore the ultrasound intensity at 370 kHz is higher than the adjacent frequencies, even though power is normalized, stronger ultrasound intensity results in stronger enhancement in the standing wave effect. According to the ultrasound safety limit given by the United States Food and Drug Administration (FDA), the ultrasound intensity applied on human should be less than 720 mW/cm^2^. If an ultrasound intensity of 700 mW/cm^2^ is applied to the PUEH, it can produce power and power density of 59.01 μW and 2.9 mW/cm^2^, respectively. This power is sufficient to support the operation of a commercialized pacemaker. The ultrasound frequency of maxima points from theoretical calculation and testing data are compared in Table 2. The frequencies from testing data almost match with the frequencies from calculation only with small deviation due to system setup and manual operation errors. For 962 kHz, there is no maxima point that matches with this frequency. It is because the PUEH barely vibrates at 962 kHz since 962 kHz is out of its operation bandwidth.

**Figure 7:**
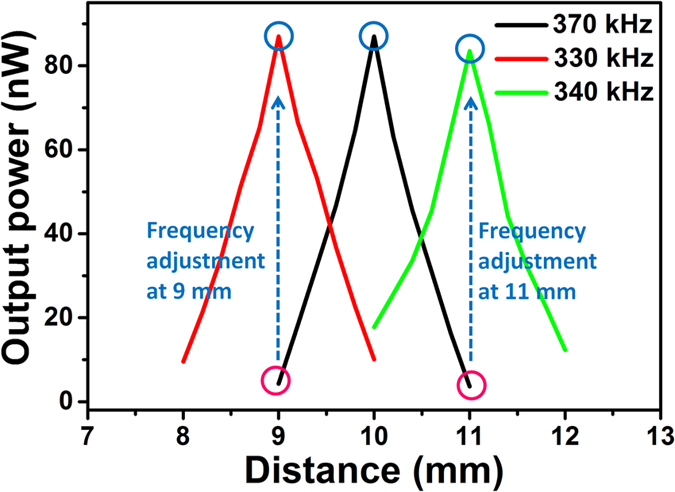
Frequency adjustment to achieve high transfer power and efficiency when 1 mm distance fluctuation happens.

Small distance fluctuation during energy transfer process is a common situation in practical implantation use due to breathing and muscle movement. This can also be overcome by using the proposed broadband PUEH. For example, if a transfer frequency of 370 kHz is adopted when the distance is 1 cm. But suddenly there is a small distance fluctuation of 1 mm due to muscle movement, then power can significantly decreases from 84.3 nW to 4.28 and 3.61 nW for 0.9 cm and 1.1 cm distance, respectively, as shown in Fig. 7. It can be observed that for both 0.9 cm and 1.1 cm distance, the power are both very low. This is because at 370 kHz, both 0.9 cm and 1.1 cm are the minima points of the standing wave. In this situation, frequency adjustment can also bring the power back to original high level. As illustrated in Fig. 7, by adjusting the frequency to 330 kHz, power at 0.9 cm distance can be significantly increased. By adjusting the frequency to 340 kHz, power at 1.1 cm can also be significantly improved. Therefore, high transfer power and efficiency can always be achieved by frequency adjustment of the PUEH for different distance or distance fluctuation in implantable applications.

**Figure 8:**
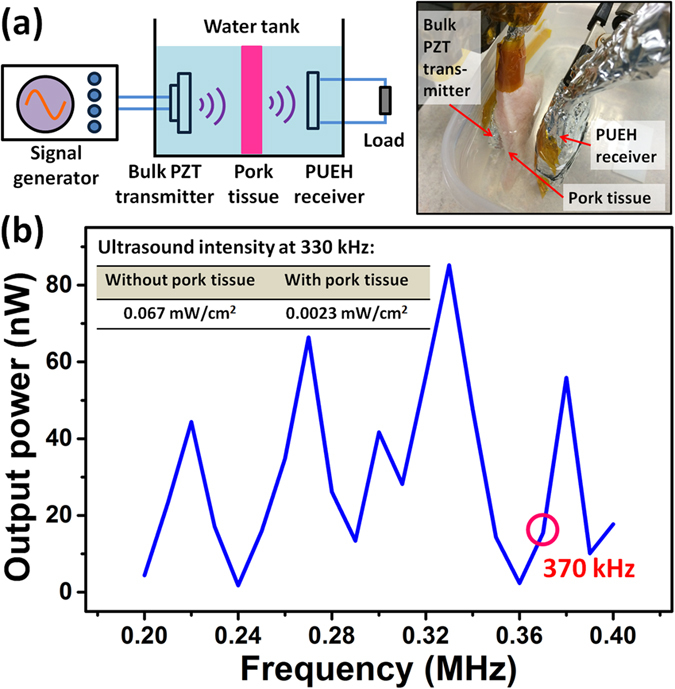
(**a**) Schematic and photograph of testing setup for power delivery through a 6 mm thick pork tissue. (**b**) Delivered power through pork tissue when ultrasound intensity of 1 mW/cm^2^ is applied.

*In vitro* test of the PUEH to transmit power through a pork tissue is shown in Fig. 8. Figure 8(a) shows the testing setup, indicating that the distance between transmitter and receiver is 2.3 cm and a 6 mm thick pork tissue is inserted in between to mimic implanted tissue. Figure 8(b) shows the normalized output power when performing frequency adjustment. When the ultrasound frequency is 370 kHz, the output power is only 16.5 nW. Then by adjusting the ultrasound frequency to 330 kHz, the output power is increased to 85.2 nW. Ultrasound intensity on the PUEH at 330 kHz without and with the presence of pork tissue is 0.067 and 0.0023 mW/cm^2^, respectively. The ultrasound intensity through a pork tissue decreases because of the impedance mismatch of pork tissue and water medium, ultrasound reflection at interface and ultrasound attenuation through the pork tissue. Even though the arrived ultrasound intensity is smaller, the PUEH still shows same energy harvesting capability since normalized power is of the same level. The ultrasound intensity on the PUEH can be increased by increasing the incoming ultrasound intensity from external ultrasound source, thus to increase output power of the PUEH for powering implantable biomedical devices. The proposed MEMS based broadband PUEH has the ability to harvest energy even from small ultrasound intensity and can be adopted as the power source for various implantable biomedical system applications.

## Discussion

In this work, a MEMS based broadband PUEH is proposed for enabling self-powered implantable biomedical devices application. The PUEH is fabricated from PZT diaphragm array with miniaturized size and optimized length to width ratio. Frequency adjustment of the PUEH can be performed to minimize the effect of standing wave for any given distances or even distance fluctuation during energy transfer process. For example, by adjusting the frequency from 250 kHz (resonant frequency of the PUEH) to 240 kHz, output power can be increased from 0.59 μW/cm^2^ to 3.75 μW/cm^2^ at distance of 1 cm and ultrasound intensity of 1 mW/cm^2^. Distance fluctuation can also be overcome by adjusting new ultrasound frequency for the fluctuated distance. The proposed MEMS based broadband PUEH has great potential to be integrated into various implantable biomedical devices for diversified applications.

## Methods

### Fabrication of the PUEH

Fabrication of the PUEH starts from a silicon-on-insulator (SOI) wafer with 10 μm device layer, 1 μm buried oxide layer (BOX), and 400 μm Si handle layer. Firstly, a 1 μm oxide layer is sputtered on top of the SOI wafer for insulation. After that, Pt (200 nm)/Ti (10 nm) layer are deposited as bottom electrode by DC magnetron sputtering. Then a 2 μm PZT layer is formed by sol-gel process. Another Pt (200 nm)/Ti (10 nm) layer are deposited on PZT as the top electrode. Next, top electrode is etched by Ar ions and PZT layer is etched by a mixture of HF, HNO_3_ and HCl. Then bottom electrode is also etched by Ar ions. Lastly, backside Si and SiO_2_ layer are etched by deep reactive-ion etching (DRIE) down to the Si device layer to release the suspending diaphragm structure.

### Characterizations and Measurements

For taking the optical microscope images, a DFC290 optical microscope (Leica) is used. For taking the SEM images, a Nova NanoSEM 230 scanning electron microscope (FEI Company) is used. For the impedance measurements of the PUEH and bulk PZT transducer, a 4294A impedance analyzer (Agilent) is used. A 33500B signal generator (Agilent) is used to generate sinusoidal signals to bulk PZT transmitter. For voltage measurement, the output signal is connected to a DSO-X3034A oscilloscope (Agilent).

## Additional Information

**How to cite this article**: Shi, Q. *et al*. MEMS Based Broadband Piezoelectric Ultrasonic Energy Harvester (PUEH) for Enabling Self-Powered Implantable Biomedical Devices. *Sci. Rep.* **6**, 24946; doi: 10.1038/srep24946 (2016).

## Supplementary Material

Supplementary Information

## Figures and Tables

**Table 1:** Tunable distance range of different maxima points.

Maxima points	1st	2nd	3rd	4th	5th	6th	7th	8th	…
Distance range (mm)	0.90 – 4.36	1.80 – 8.71	2.70 – 13.07	3.60 – 17.42	4.50 – 21.78	5.40 – 26.13	6.30 – 30.49	7.20 – 34.84	…

**Table 2:** Ultrasound frequency of maxima points from theoretical calculation and testing data at 1 cm transfer distance.

Calculation (kHz)	148	222	296	370	444	518	592	666	740	814	888	962
Testing data (kHz)	130	240	290	370	410	540	570	650	730	780	910	\
