# Supplementary material for: MEMS Based Broadband Piezoelectric Ultrasonic Energy Harvester (PUEH) for Enabling Self-Powered Implantable Biomedical Devices
Source: Sci Rep. 2016 Apr 26;6:24946. doi: 10.1038/srep24946 (PMC4844957; doi:10.1038/srep24946)
Supplement: Supplementary Information [file srep24946-s1.pdf]

Supplementary Information

MEMS Based Broadband Piezoelectric Ultrasonic Energy Harvester (PUEH) for Enabling Self-Powered Implantable Biomedical Devices

Qiongfeng Shi[1,2], Tao Wang[1,2] and Chengkuo Lee[1,2*]

[1] Department of Electrical and Computer Engineering, National University of Singapore, 4 Engineering Drive 3, Singapore 117576

[2] Center for Intelligent Sensors and MEMS, National University of Singapore, 4 Engineering Drive 3, Singapore 117576

*Corresponding Author: Chengkuo Lee
E-mail: elelc@nus.edu.sg

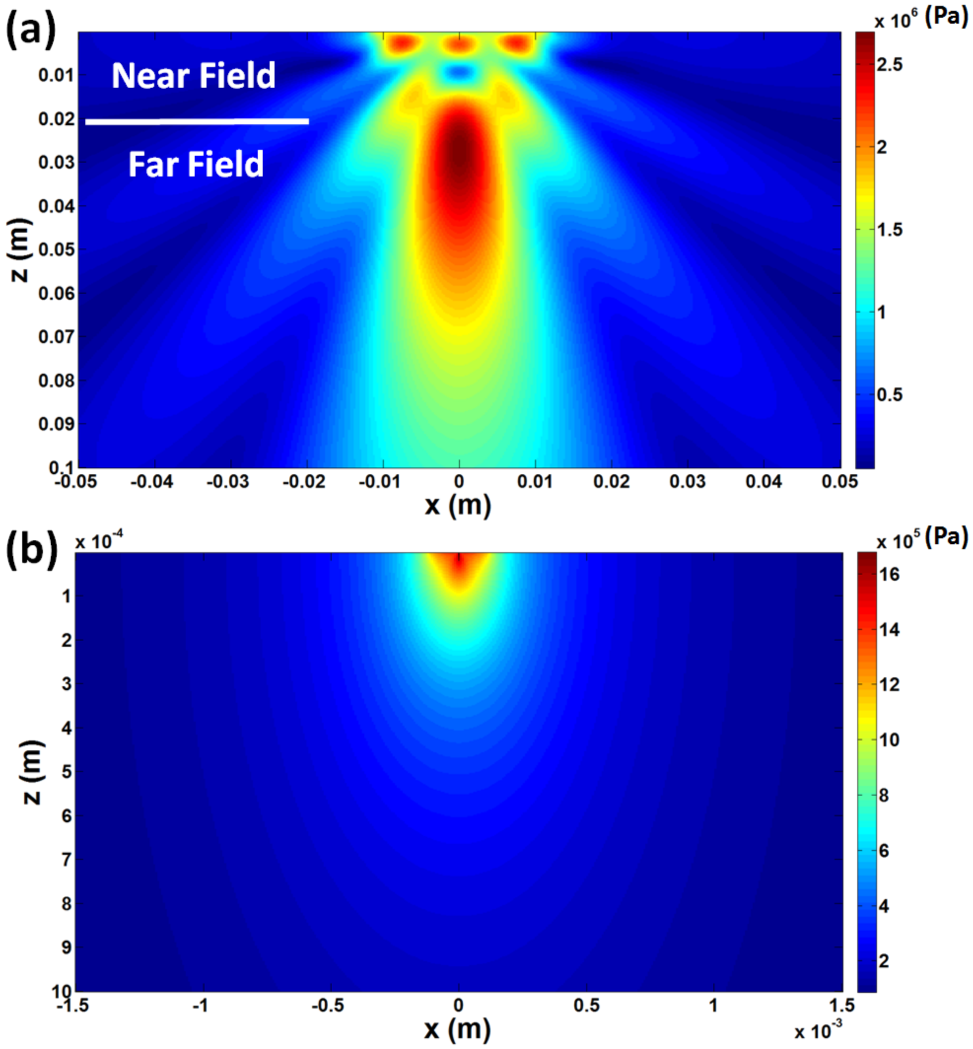

Figure S1: (a) Ultrasound pressure pattern of the bulk lead zirconate titanante (PZT) transducer (diameter 25 mm) by Transducer Array Calculation GUI (Karlsruhe Institute of Technology), showing the near field regime is within 2 cm. (b) Ultrasound pressure pattern of the proposed piezoelectric ultrasonic energy harvester (PUEH), showing the near field regime is within 100 µm range. Input sinusoidal signal is 200 kHz, 2 Vpp.

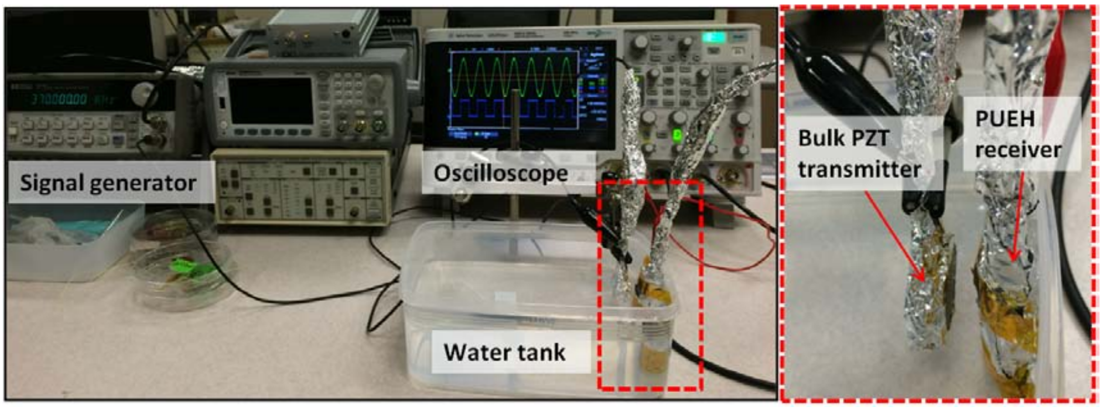

Figure S2: Photograph of testing setup of the power transfer system for voltage and power measurement.

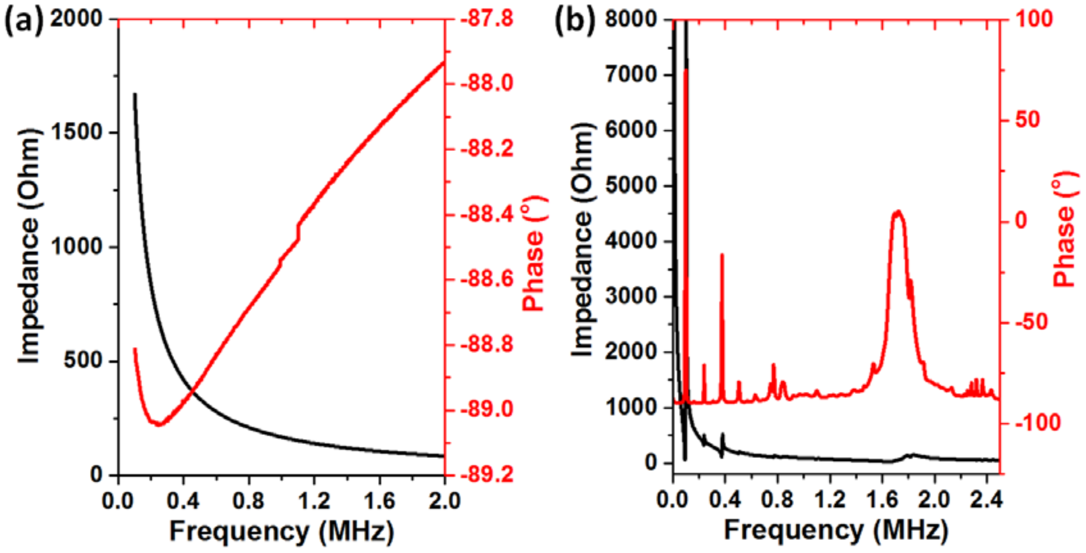

Figure S3: Impedance of (a) the PUEH and (b) bulk PZT transducer measured in water with respect to frequency.

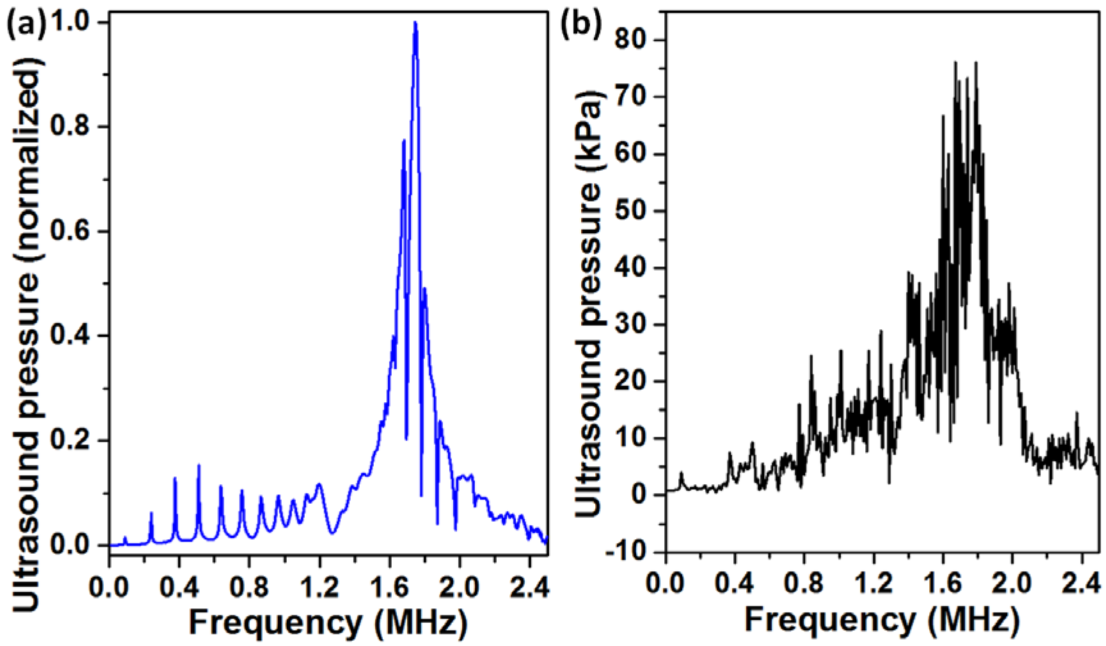

Figure S4: (a) Simulation and (b) testing results of the generated ultrasound pressure by bulk PZT transducer at 1 cm distance.
